# Fatty Acids as Aminoglycoside Antibiotic Adjuvants Against *Staphylococcus aureus*

**DOI:** 10.3389/fmicb.2022.876932

**Published:** 2022-05-12

**Authors:** Sunyoung Park, Jin-Hyung Lee, Yong-Guy Kim, Liangbin Hu, Jintae Lee

**Affiliations:** ^1^School of Chemical Engineering, Yeungnam University, Gyeongsan, South Korea; ^2^School of Food & Biological Engineering, Shaanxi University of Science and Technology, Xi'an, China

**Keywords:** aminoglycoside antibiotics, biofilm, fatty acids, myristoleic acid, *S. aureus*

## Abstract

Fatty acids have diverse functions in the vast majority of cells. At high doses, they act as antimicrobials while, at low doses, they exhibit antibiofilm and antivirulence activities. In this study, the synergistic antibacterial and antibiofilm activities of 30 fatty acids and 11 antibiotics were investigated against methicillin-sensitive and methicillin-resistant *Staphylococcus aureus* strains. Of the 15 saturated and 15 unsaturated fatty acids examined, 16 enhanced the antibacterial activity of tobramycin. Combinatorial treatment with myristoleic acid (the most active) at 10 μg/ml and tobramycin at 10 μg/ml decreased cell survival by >4 log as compared with tobramycin treatment alone. Notably, aminoglycoside antibiotics, such as tobramycin, kanamycin, gentamicin, and streptomycin exhibited antimicrobial synergy with myristoleic acid. Co-treatment with myristoleic acid and antibiotics markedly decreased biofilm formation. Interestingly, co-treatment with tobramycin and myristoleic acid induced a reduction in *S. aureus* cell size. These results suggest that fatty acids, particularly myristoleic acid, can be used as aminoglycoside antibiotic adjuvants against recalcitrant *S. aureus* infections.

## Introduction

Fatty acids (FAs), both free and in complex lipids, are ubiquitously distributed in animals, plants, and microbes. FAs are carboxylic acids with a saturated or unsaturated aliphatic chain and have diverse functions in cells, for example, they act as cell membrane structural components, suppliers of energy, and signaling molecules (de Carvalho and Caramujo, 2018). Recently, FAs were suggested to be potential antimicrobial agents at high doses (Desbois and Smith, 2010) and to act as antibiofilm and antivirulence agents at low doses (Kumar et al., 2020). The mechanism responsible for their antimicrobial activities presumably involves cell membrane disruption due to their amphipathic characters (Desbois and Smith, 2010), and the mechanism responsible for their antivirulence activities probably involves interference with bacterial signaling (Kumar et al., 2020). However, details of the molecular mechanisms involved remain to be revealed. Several reports have shown some FAs enhance the antibacterial activities of antibiotics (Hess et al., 2014; Chan et al., 2015; Casillas-Vargas et al., 2021; Kim et al., 2021a). However, no comprehensive study has been undertaken to compare the synergistic antibacterial and antibiofilm activities of a series of FAs.

*Staphylococcus aureus* is a major pathogen of acute and chronic infections and is responsible for worldwide outbreaks of nosocomial infections (Lowy, 1998). *Staphylococcus aureus* often exhibits antibiotic resistance, and its biofilms on medical devices and host surfaces play a critical role in antibiotic tolerance (Stewart and Costerton, 2001; Seo et al., 2021). Lauric acid (Hess et al., 2014), linoleic and oleic acids (Chan et al., 2015), and myristic acid (Kim et al., 2021b) have been reported to act synergistically with antibiotics to enhance antibacterial efficacy against *S. aureus*. Therefore, we investigated the effects of FAs and antibiotic co-treatments on the antibacterial and antibiofilm activities of *S. aureus* strains.

In this study, 30 natural FAs (two short-chain, seven medium-chain, 16 long-chain, and five very long-chain) were initially screened for their bactericidal activities when co-administered with tobramycin against *S. aureus*. The synergistic antibacterial and antibiofilm activities of the most active FA, myristoleic acid, were confirmed in combination with four aminoglycoside antibiotics, namely, tobramycin, kanamycin, gentamicin, and streptomycin. Conventional microscopy and scanning electron microscopy (SEM) were used to observe changes in biofilm formation by *S. aureus*.

## Materials and Methods

### Strains, Chemicals, and Culture Materials

In this study, we used methicillin-sensitive *S. aureus* (MSSA; ATCC 6538) and methicillin-resistant *S. aureus* (MRSA; MW2) strains. All experiments were performed at 37°C in Luria-Bertani medium (Silber et al., 2020). Thirty FAs, namely butanoic acid (C4:0), pentanoic acid (C5:0), hexanoic acid (C6:0), heptanoic acid (C7:0), octanoic acid (C8:0), nonanoic acid (C9:0), decanoic acid (C10:0), undecanoic acid (C11:0), lauric acid (C12:0), myristic acid (C14:0), myristoleic acid (C14:1), palmitic acid (C16:0), palmitoleic acid (C16:1 ω-5), heptadecanoic acid (C17:0), stearic acid (C18:0), vaccenic acid (C18:1 ω-7), oleic acid (C18:1 ω-9, *cis*), elaidic acid (C 18:1 ω-9, *trans*), petroselinic acid (C18:1 ω-12), linoleic acid (C18:2 ω-6), conjugated linoleic acid (C18:2 ω-6), α-linolenic acid (C18:3 ω-3), γ-linolenic acid (C18:3 ω-6), arachidonic acid (C20:4 ω-6), eicosapentaenoic acid (C20:5 ω-3), behenic acid (C22:0), erucic acid (C22:1 ω-9), docosahexaenoic acid (C22:6 ω-3), tricosanoic acid (C23:0), and nervonic acid (C24:1 ω-9) were purchased from Sigma-Aldrich (St. Louis, United States) or TCI Co. (Tokyo, Japan). All FAs except butanoic acid were dissolved in dimethyl sulfoxide (DMSO); butanoic acid was dissolved in water. DMSO at 0.1% v/v, which had no effect on bacterial growth or biofilm formation, was used as a control. Antibiotics *viz.* tobramycin, kanamycin, streptomycin, amoxicillin, vancomycin, tetracycline, erythromycin, rifampicin, gentamicin, ciprofloxacin, and methicillin were obtained from Sigma-Aldrich.

Planktonic cell growths and turbidities were measured at 600 nm using an Optizen 2120 UV spectrophotometer (Mecasys Co. Ltd., Daejeon, Korea). MIC was defined as the lowest concentration that visually inhibited the growth of planktonic cells, and MICs were also confirmed by colony counting and droplet assay modified from previous publication (Sieuwerts et al., 2008).

### Co-treatments With Fatty Acids and Antimicrobial Agents

The combinatorial efficacies of FAs and antimicrobial agents were analyzed using the two *S. aureus* strains. Briefly, overnight cultures at an initial turbidity of OD 0.05 at 600 nm were inoculated into proper culture media (final volume 1,000 μl) with antibiotics and/or FAs. Cells were incubated with shaking for 1 h at 37°C, and cell mixtures were diluted serially in PBS (phosphate-buffered saline) and plated on LB agar plates. CFUs were determined by colony counting after incubation for 24 h at 37°C. Experiments were performed using two independent cultures in quadruplicate.

### Crystal Violet Biofilm Assay

Biofilm formation by the two *S. aureus* strains was quantified in 96-well polystyrene plates (SPL Life Sciences, Korea), as previously described (Lee et al., 2019). Briefly, cells were inoculated into LB broth (300 μl) at an initial turbidity of 0.05 (3.5 × 10^9^ CFU/ml) at 600 nm and FAs were added at different concentrations and incubated for 24 h at 37°C in a static state. To quantify biofilm formation, cell cultures were rinsed three times with water (300 μl), and then biofilms were tinted with 0.1% crystal violet (300 μl) for 20 min, and dissolved in 300 μl of 95% ethanol. Absorbances were measured at 570 nm (OD_570_) using a Multiskan EX microplate reader (Thermo Fisher Scientific, Waltham, USA). Static biofilm formation results are the averages of six replicate wells of two representative independent experiments.

### Biofilm Observations by Live Imaging Microscopy and SEM

To analyze biofilms, they were produced, as mentioned above, over 24 h at 37°C. After incubation, planktonic cells were discarded by gentle washing with distilled water three times and biofilms were analyzed by live imaging microscopy using the iRiS™ Digital Cell Imaging System (Logos BioSystems, Anyang, Korea) at different magnifications. Biofilm images were regenerated as color-coded 2D and 3D pictures using ImageJ.^1^ *Staphylococcus aureus* biofilms on nylon filter membranes were also examined by SEM, as previously described (Kim et al., 2016). Briefly, a nylon filter membrane (Merck Millipore, Burlington, United States) was cut into 0.4 cm × 0.4 cm pieces, autoclaved, and placed in each well of 96-well plates containing cell culture and incubated with or without myristoleic acid and/or tobramycin for 24 h at 37°C. Biofilm cells on nylon membranes were washed with PBS, fixed with glutaraldehyde (2.5%) and formaldehyde (2%) for 24 h, post-fixed with OsO_4_ (Osmium tetroxide), and dehydrated using an ethanol series (50%, 70%, 80%, 90%, 95%, and 100%) and isoamyl acetate. After critical-point drying and sputter coating, cells on membranes were imaged using an S-4800 scanning electron microscope (Hitachi, Tokyo, Japan) at 15 kV.

### Statistical Analysis

Replication numbers for assays are provided above and results are expressed as means ± standard deviations. The statistical analysis was performed by one-way ANOVA followed by Dunnett’s test using SPSS Ver. 23 (SPSS Inc., Chicago, United States). The values of *p* < 0.05 were considered as significant, and asterisks indicate significant differences between treated and untreated samples.

## Results

### Combinatorial Antimicrobial Activities of Fatty Acids

**Figure 1:**
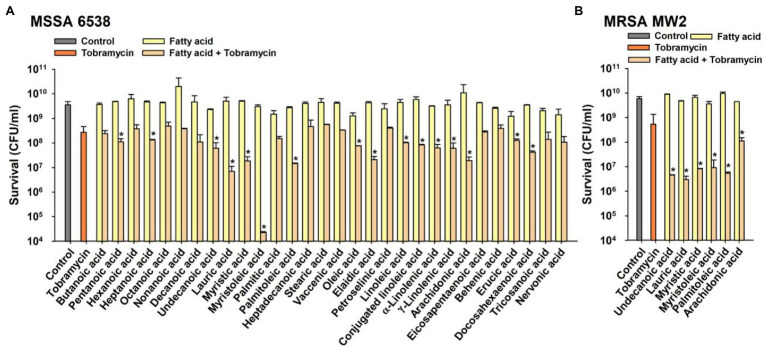
Combinatorial antimicrobial activities of fatty acids and tobramycin against *Staphylococcus aureus*. Cell survivals were measured after treating MSSA 6538 **(A)** or MRSA MW2 **(B)** with tobramycin (10 μg/ml) and/or a FA (10 μg/ml) for 1 h. *p < 0.05 vs. tobramycin-treated controls. Control; untreated control.

The combinatorial antimicrobial activities of the 30 FAs (15 saturated and 15 unsaturated FAs) at 10 μg/ml were initially investigated against a methicillin-sensitive *S. aureus* strain (MSSA; ATCC 6538) in combination with the aminoglycoside tobramycin. Tobramycin treatment alone at a concentration of 10 μg/ml in 1 h decreased cell survival by 92%, whereas treatment with any of the FAs alone at the same concentration did not affect cell survival (Figure 1). Interestingly, co-treatment with tobramycin and FAs showed some synergistic antimicrobial effects with widely different efficiencies. Sixteen FAs, namely pentanoic (C5:0), heptanoic (C6:0), undecanoic (C11:0), lauric (C12:0), myristic (C14:0), myristoleic (C14:1), palmitoleic (C16:1 ω-5), oleic (C18:1 ω-9, *cis*), elaidic (C18:1 ω-9, *trans*), linoleic (C18:2 ω-6), conjugated linoleic (C18:2 ω-6), α-linolenic (C18:3 ω-3), γ-linolenic (C18:3 ω-6), arachidonic (C20:4 ω-6), erucic (C22:1 ω-9), and docosahexaenoic acid (C22:6 ω-3) significantly decreased cell survival as compared with tobramycin treatment alone (Figure 1A). Most notably, myristoleic acid plus tobramycin decreased cell survival by >5 log as compared with 1 log reduction by tobramycin alone. The synergistic antimicrobial efficacies of the six active FAs, namely, undecanoic, lauric, myristic, myristoleic, palmitoleic, and arachidonic acid, were also observed on a methicillin-resistant *S. aureus* strain (MRSA; MW2) and reduced cell survival by >1–3 log vs. tobramycin alone (Figure 1B). Hence, in subsequent studies, we focused on the effects of the most active FA, myristoleic acid, on both *S. aureus* strains.

The MICs of the 16 active FAs against MSSA ATCC 6538 are shown in Table 1. While the MICs of most FAs were > 400 μg/ml, the MICs of undecanoic, myristoleic, and linoleic acid were 200, 100, and 200 μg/ml, respectively. These results suggest that the antibacterial synergistic effect of FAs at 10 μg/ml was due to synergism between antibiotics and FAs but not to their antimicrobial activities.

**Table 1:** MICs of fatty acids for *Staphylococcus aureus* ATCC 6538.

Lipid number	Fatty acids	MIC (μg/ml)
C5:0	Pentanoic acid	>400
C7:0	Heptanoic acid	>400
C11:0	Undecanoic acid	200
C12:0	Lauric acid	400
C14:0	Myristic acid	>400
C14:1	Myristoleic acid	100
C16:1 ω-5	Palmitoleic acid	400
C18:1 ω-9, *cis*	Oleic acid	>400
C18:1 ω-9, *trans*	Elaidic acid	>400
C18:2 ω-6	Linoleic acid	200
C18:2 ω-6	Conjugated linoleic acid	>400
C18:3 ω-3	α-Linolenic acid	>400
C18:3 ω-6	γ-Linolenic acid	>400
C20:4 ω-6	Arachidonic acid	>400
C22:1 ω-9	Erucic acid	>400
C22:6 ω-3	Docosahexaenoic acid	>400

*Staphylococcus aureus* cells were inoculated into LB broth at an initial turbidity of 0.05 at 600 nm and incubated in 96-well plates containing each of the 16 tested fatty acids for 24 h at 37°C. MIC was defined as the lowest concentration that inhibited microbial growth as assessed by spectrophotometry (620 nm), CFU counting and droplet assay.

### Antibacterial Synergy Between Myristoleic Acid and Aminoglycosides

To investigate which antibiotics, exhibit synergism with FAs, *S. aureus* was treated with 10 common antibiotics, namely kanamycin, streptomycin, amoxicillin, vancomycin, tetracycline, erythromycin, rifampicin, gentamicin, ciprofloxacin, and methicillin up to 200 μg/ml for 1 h. Three aminoglycosides, namely, kanamycin, gentamicin, and streptomycin significantly decreased cell survival, but the other seven antibiotics did not, indicating that these three had bactericidal effects (Supplementary Figure 1). Interestingly, unlike other antibiotics, tobramycin, kanamycin, gentamicin, and streptomycin are all aminoglycoside antibiotics.

**Figure 2:**
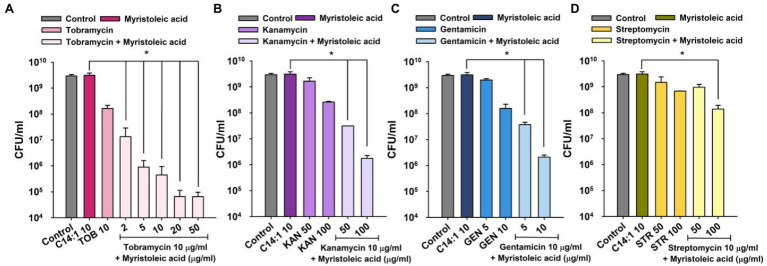
Synergistic antimicrobial activities of myristoleic acid and aminoglycoside antibiotics against *Staphylococcus aureus*. Cell survivals were measured 1 h after treating MSSA 6538 with myristoleic acid (10 μg/ml) and/or tobramycin **(A)**, kanamycin **(B)**, gentamicin **(C)**, or streptomycin **(D)**. *p < 0.05 vs. myristoleic acid-treated controls. TOB; tobramycin, KAN; kanamycin, GEN; gentamicin, STR; streptomycin, C14:1; myristoleic acid, Control; untreated control.

As was expected, myristoleic acid in combination with kanamycin, gentamicin, or streptomycin dose-dependently decreased cell survival as compared with tobramycin treatment alone (Figure 2). For example, myristoleic acid (10 μg/ml) plus kanamycin (50 or 100 μg/ml) or gentamicin (5 or 10 μg/ml) decreased cell survival by >2 log while the synergistic effect of myristoleic acid was less when administered with streptomycin.

### Synergistic Antibiofilm Effects of Myristoleic Acid and Aminoglycoside Antibiotics

**Figure 3:**
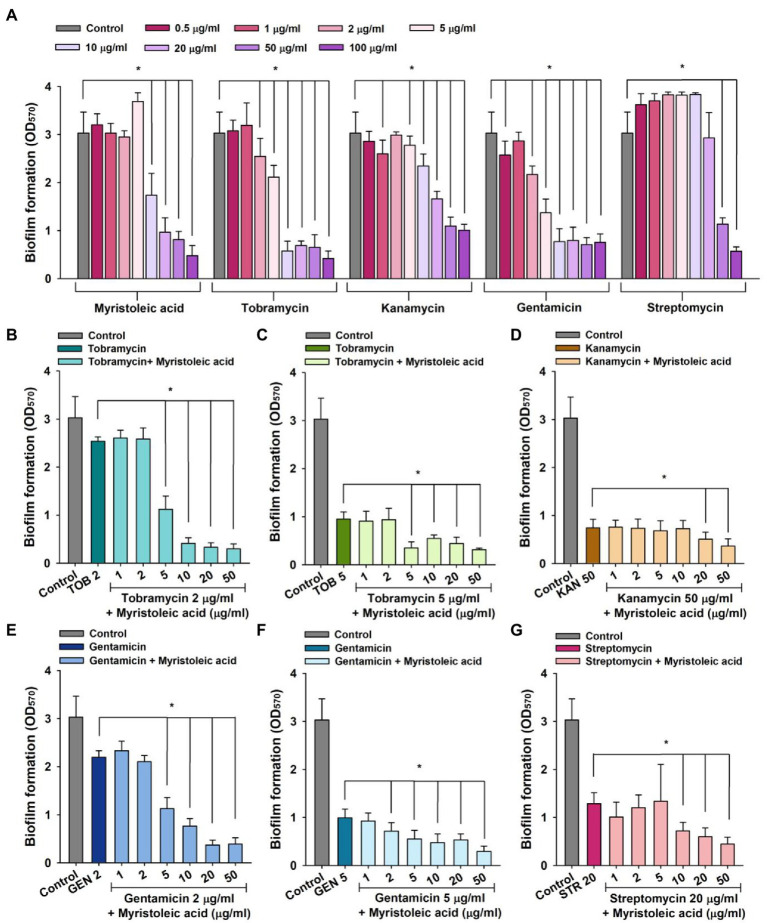
*Staphylococcus aureus* biofilm inhibition by antibiotics in the presence or absence of fatty acids. Biofilm inhibitions by myristoleic acid, tobramycin, kanamycin, gentamicin, or streptomycin **(A)** were determined after incubation for 24 h in 96-well plates at 37°C under static conditions. Combinatorial antibiofilm activities of tobramycin **(B,C)**, kanamycin **(D)**, gentamicin **(E,F)**, or streptomycin **(G)** in the presence of myristoleic acid were measured after incubation under identical conditions. Error bars indicate SD. *p < 0.05 vs. untreated controls **(A)**. *p < 0.05 vs. antibiotic-treated controls **(B–G)**. TOB; tobramycin, KAN; kanamycin, GEN; gentamicin, STR; streptomycin, Control; untreated control.

*Staphylococcus aureus* biofilm formation plays important roles in antimicrobial resistance and a variety of device-related infections (Stewart and Costerton, 2001), and sub-inhibitory concentrations of aminoglycosides often induce biofilm formation (Hoffman et al., 2005). Hence, we investigated the antibiofilm activities of tobramycin, kanamycin, gentamicin, and streptomycin alone and in combination with myristoleic acid. Treatment of MSSA ATCC 6538 with myristoleic acid alone dose-dependently reduced biofilm formation (Figure 3A), which is in agreement with a recent study which reported that myristoleic acid inhibited biofilm formation by *Cutibacterium acnes* alone or with *S. aureus* (Kim et al., 2021a). Low concentrations of myristoleic acid, tobramycin, and streptomycin slightly increased biofilm formation by *S. aureus*, whereas high doses decreased biofilm formation (Figure 3A). For example, streptomycin at 1 or 10 μg/ml increased *S. aureus* biofilm formation by 22% and 26%, respectively (Figure 3A).

To study the antibiofilm efficacies of myristoleic acid and antibiotic co-treatments, we administered antibiotics at concentrations that reduced biofilm formation by 16 to 75%. For example, tobramycin at 2 or 5 μg/ml, kanamycin at 50 μg/ml, gentamicin at 2 or 5 μg/ml, and streptomycin at 20 μg/ml were subjected to combinatorial assays.

Co-treatment with myristoleic acid and antibiotics enhanced antibiofilm activities. For example, tobramycin alone at 2 μg/ml reduced biofilm formation by 16%, whereas treatment with tobramycin (2 μg/ml) and myristoleic acid (5 μg/ml) reduced biofilm formation by 63% (Figure 3B). Similar results were obtained for tobramycin at 5 μg/ml (Figure 3C). Gentamicin alone at 2 μg/ml reduced biofilm formation by 28% and treatment with gentamicin (2 μg/ml) and myristoleic acid (5 μg/ml) reduced biofilm formation by 63% (Figure 3E). Similar results were also obtained for gentamicin at 5 ug/ml (Figure 3F). However, myristoleic acid had a smaller synergistic effect on the antibiofilm activities of kanamycin (Figure 3D) and streptomycin (Figure 3G). Fractional inhibitory concentration indices (FICI) for the antibiofilm activity were calculated to check the synergistic effects of myristoleic acid/antibiotic combinations. FICI values of tobramycin, gentamicin, kanamycin, and streptomycin were 0.2–0.3, 0.3–0.52, 1.5, and 0.6, respectively. Hence, combinatory effects were synergistic for tobramycin and gentamicin, additive for streptomycin, and indifferent for kanamycin.

**Figure 4:**
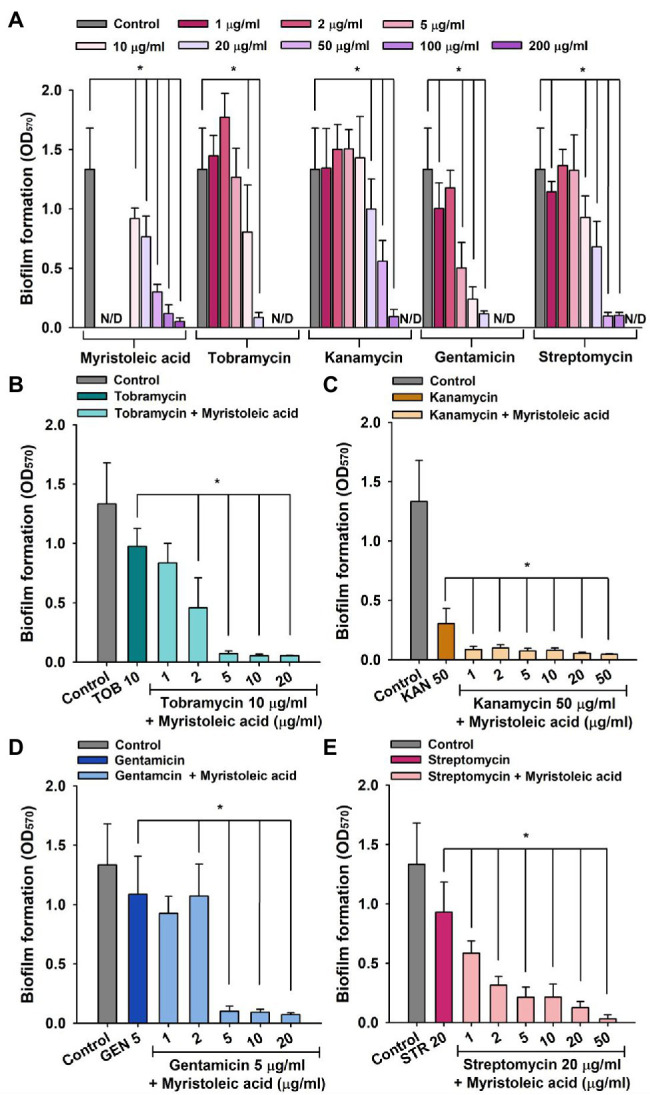
MRSA biofilm inhibition by antibiotics in the presence or absence of fatty acid. Biofilm inhibitions by myristoleic acid, tobramycin, kanamycin, gentamicin, and streptomycin **(A)** were measured after incubating in 96-well plates for 24 h at 37°C under static conditions. Antibiofilm activities of tobramycin **(B)**, kanamycin **(C)**, gentamicin **(D)**, or streptomycin **(E)** plus myristoleic acid were measured after incubation under identical conditions. Error bars indicate standard deviations. *p < 0.05 vs. untreated controls **(A)**. *p < 0.05 vs. antibiotic-treated controls **(B–E)**. TOB; tobramycin, KAN; kanamycin, GEN; gentamicin, STR; streptomycin, Control; untreated control. N/D indicates “Not Determined.”

In addition, combinatory antibiofilm efficacy trends of myristoleic acid and antibiotics against MRSA MW2 and MSSA ATCC 6538 were found to be similar, although MRSA MW2 produced less biofilm than MSSA ATCC 6538 (Figure 4).

### Microscopic Observations of *Staphylococcus aureus* Biofilm Inhibition by Myristoleic Acid and/or Tobramycin

**Figure 5:**
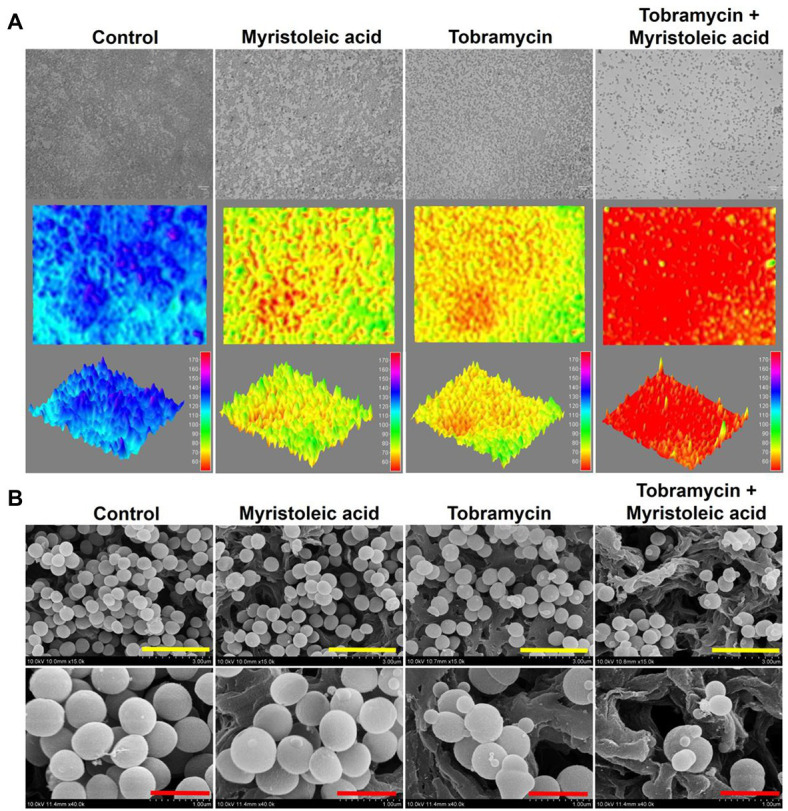
Biofilm cells of *Staphylococcus aureus* grown in the presence or absence of myristoleic acid and/or tobramycin. Biofilm inhibitions by myristoleic acid (20 μg/ml) and/or tobramycin (5 μg/ml) were determined after culture for 24 h under static conditions. Re-created color-coded 2D and 3D images of *Staphylococcus aureus* biofilms **(A)**. SEM images of *Staphylococcus aureus* biofilms formed in the presence or absence of myristoleic acid (20 μg/ml) and/or tobramycin (5 μg/ml; **B**). Yellow and red scale bars represent 3 and 1 μm, respectively. Control; untreated control.

Biofilm inhibition was analyzed using a live cell imaging system and by SEM. 3-D color mesh plots showed that tobramycin at 5 μg/ml or myristoleic acid at 20 μg/ml inhibited biofilm formation by *S. aureus*, but that in combination they had a markedly greater effect (Figure 5A). SEM analysis confirmed the inhibitory effect of myristoleic acid along with tobramycin and also showed the shape of *S. aureus* biofilm cells on the surface of nylon filter membrane. Treatment of myristoleic acid, tobramycin, or their combination markedly reduced the numbers of *S. aureus* cells. Tobramycin at 5 μg/ml or myristoleic acid at 20 μg/ml did not cause any surface defects or membrane damages. Interestingly, tobramycin or myristoleic acid produced few small cells, but in combination they produced many smaller cells (Figure 5B). Taken together, myristoleic acid co-treatment enhanced the antibiofilm effects of antibiotics on *S. aureus*.

## Discussion

Since penicillin was discovered in 1940, various antibiotics have been used as treatments of choice to treat infectious diseases. However, the overuse of antibiotics has caused the worldwide emergence of drug-resistant pathogens. Also, bacterial biofilms are tolerant of conventional antimicrobial therapeutics and the host immune system. Hence, alternative approaches are needed to control drug resistance and biofilm formation, and antibiotic adjuvants provide one such alternative. This study presents that several FAs, particularly myristoleic acid, enhance the bactericidal activities of four aminoglycoside antibiotics, namely, kanamycin, gentamicin, streptomycin, and tobramycin against *S. aureus* and that combinatory treatment of myristoleic acid and these antibiotics significantly decreases biofilm formation by *S. aureus*, which suggests the possible use of myristoleic acid as an antibiotic adjuvant.

Antibiotic adjuvants include: (1) anti-resistance drugs such as β-lactamase inhibitors pump inhibitors, membrane permeabilizers, and biofilm inhibitors; (2) antivirulence drugs, such as quorum-sensing inhibitors, toxin inhibitors, and secretion system inhibitors; (3) host-directed therapies such as innate immune system agonists and immunomodulatory peptides; and (4) other therapies such as phage, probiotic, and oral rehydration therapies (Gill et al., 2015). Various synthetic and natural compounds such as prazolopyridazine derivatives (Schaenzer et al., 2018), isoflavonoids (Abreu et al., 2017), plant extracts (Abreu et al., 2016), cationic peptides (Geitani et al., 2019), cannabidiol (Wassmann et al., 2022), marine bisindole alkaloid (Campana et al., 2020), cinnamonitrile (Speri et al., 2019), amine oxide alkyl derivatives (Fagnani et al., 2021), and brominated carbazoles (Berndsen et al., 2022) have been recently proposed to be used as antibiotic adjuvants against *S. aureus* strains.

The FAs identified in this study are potential anti-resistance agents since they enhanced antibiotic efficacy and inhibited biofilm formation. Recent studies have suggested that FAs could be used as antibiotic adjuvants (Kim et al., 2018; Kumar et al., 2020; Yuyama et al., 2020). In particular, several FAs, such as lauric acid plus gentamicin or streptomycin (but not ampicillin or vancomycin; Hess et al., 2014), linoleic or oleic acids with erythromycin (Chan et al., 2015), and lauric or myristic acids with the aminoglycosides gentamicin, kanamycin, or tobramycin (Kim et al., 2021b) showed synergistic antibacterial efficacy. However, the action mechanisms responsible for this antibacterial synergy conferred by FAs are unknown. It has long been reported that FAs at high concentrations have antimicrobial activities, probably because they alter cell membrane permeability, disrupt membranes, and cause leakage of intracellular metabolites (Desbois and Smith, 2010). However, a few reasonable mechanisms have been suggested to explain the antibacterial synergistic effects of FAs. In particular, it was suggested that the synergistic antibacterial activities of linoleic and oleic acids on the activity of the macrolide erythromycin were probably due to efflux MsrA pump interference in *S. aureus* (Chan et al., 2015). In another study, it was speculated that the synergistic antibiofilm effect of lauric acid on the antibiofilm effects of gentamicin and streptomycin were due to the facilitation of biofilm matrix penetration due to surfactant effects (Hess et al., 2014).

Interestingly, in the present study, myristoleic acid alone at low doses did not exhibit antibacterial activity but had synergistic effects on the bactericidal activities of four aminoglycosides (Figure 2). Although speculative, myristoleic acid, like linoleic and oleic acids, may target efflux MsrA pump and possibly interfere with protein synthesis *via* the 30S ribosomal subunit, which is the action mechanism of aminoglycosides. Intriguingly, small size *S. aureus* cells were produced by treating *S. aureus* with tobramycin and/or myristoleic acid (Figure 5). Furthermore, it was mentioned in a previous report that small *S. aureus* cells were produced by treatment with the antibiotic nitrofuran (Koike et al., 1974), whereas long or large cells were often observed after the inhibition of cell division *via* the major cell division protein FtsZ (Silber et al., 2020). Hence, it is possible that low doses of antibiotics plus myristoleic acid disturb the regulation of cell division in *S. aureus*. In addition, we suggest that since aminoglycosides exhibit bactericidal activity against most Gram-negative bacilli, our findings may be applicable to other Gram-negative bacteria. Recently, several FAs were reported to exhibit antibiofilm activity (Kim et al., 2018; Kumar et al., 2020; Yuyama et al., 2020). In. *S. aureus*, oleic acid, *cis*-11-eicosenoic acid, and two long chain omega-3 FAs inhibited *S. aureus* biofilm formation and hemolytic activity by suppressing the alpha-hemolysin *hla* gene (Lee et al., 2017; Kim et al., 2018). Kim et al. reported myristoleic acid inhibited the formations of *C. acnes* and *S. aureus* biofilms and mixed *C. acnes*/*S. aureus* biofilms, and that lauric and myristic acids inhibited polymicrobial biofilm formation by *S. aureus*, *Escherichia coli* O157:H7, and *Candida albicans* (Kim et al., 2021a). These observations indicate that FAs, such as myristoleic acid, could be used as antibiotic adjuvants with antibiotics to target many species of microbes.

## Data Availability Statement

The original contributions presented in the study are included in the article/Supplementary Material, further inquiries can be directed to the corresponding author.

## Author Contributions

SP and JL: conceptualization, data curation, writing of the manuscript, and visualization. SP, J-HL, and Y-GK: methodology, validation, formal analysis, and investigation. SP and J-HL: software. JL: resources. J-HL and JL: project administration. All authors contributed to the article and approved the submitted version.

## Funding

This research was supported by the Basic Science Research Program of the National Research Foundation of Korea (NRF) funded by the Ministry of Education (2021R1I1A3A04037486 to J-HL), the NRF funded by the Korea government (MSIT; 2021R1A2C1008368), and by the Priority Research Center Program of the NRF funded by the Ministry of Education (2014R1A6A1031189).

## Conflict of Interest

The authors declare that the research was conducted in the absence of any commercial or financial relationships that could be construed as a potential conflict of interest.

## Publisher’s Note

All claims expressed in this article are solely those of the authors and do not necessarily represent those of their affiliated organizations, or those of the publisher, the editors and the reviewers. Any product that may be evaluated in this article, or claim that may be made by its manufacturer, is not guaranteed or endorsed by the publisher.

## Footnotes

^1^ https://imagej.nih.gov/ij/index.html
