# Supplementary material for: Fatty Acids as Aminoglycoside Antibiotic Adjuvants Against Staphylococcus aureus
Source: Front Microbiol. 2022 May 12;13:876932. doi: 10.3389/fmicb.2022.876932 (PMC9133387; doi:10.3389/fmicb.2022.876932)
Supplement: Supplementary file 1 [file Image_1.pdf]

# *Supplementary Material*

## 1 Supplementary Figures

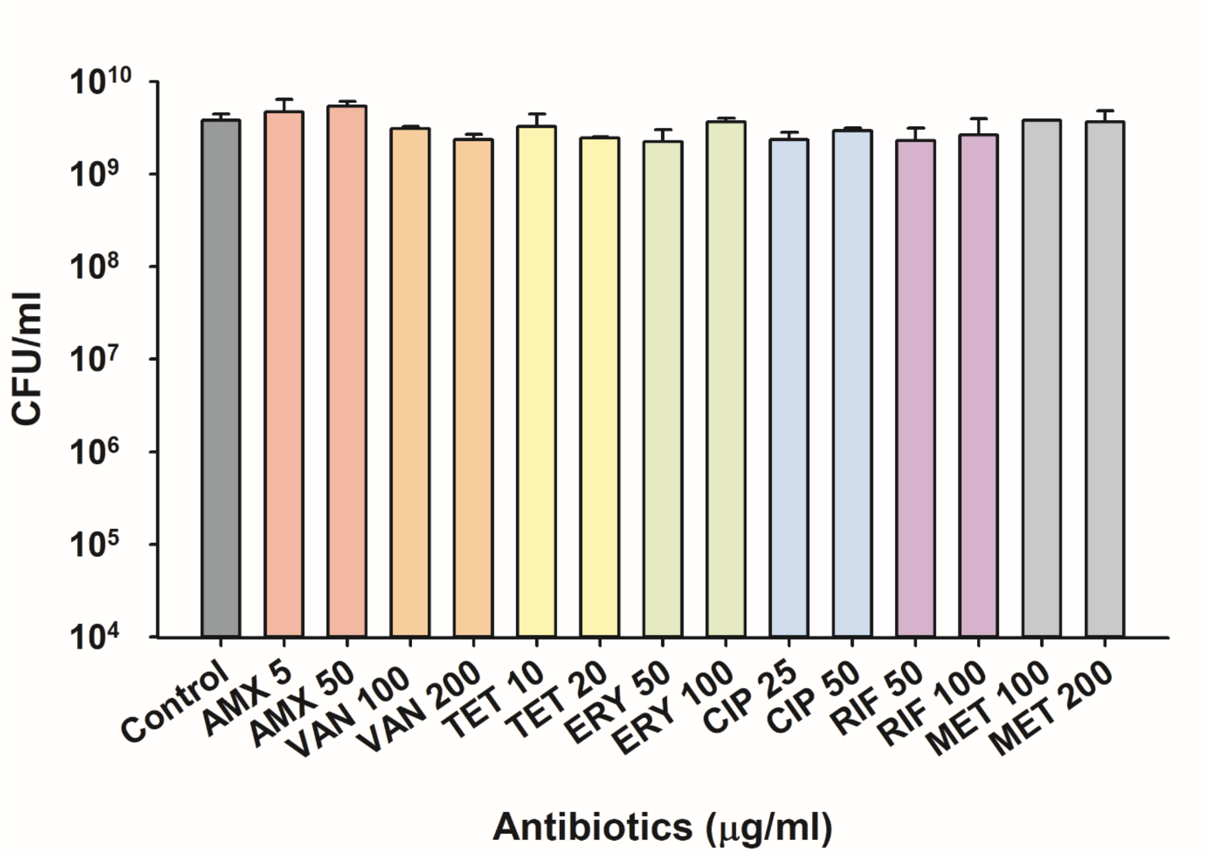


**Supplementary Figure 1.** Antimicrobial activities of antibiotics against S. aureus. S. aureus cell survivals were measured after treatment with 7 antibiotics for 1 h. AMX; amoxicillin, VAN; vancomycin, TET; tetracycline, ERY; erythromycin, CIP; ciprofloxacin, RIF; rifampicin, MET; methicillin, Control; untreated control.
